# Cataract services: ensuring access for everyone

**Published:** 2014

**Authors:** Robert Lindfield

**Affiliations:** Clinical Lecturer: The Disability and Eye Health Group, London, UK Robert.Lindfield@Lshtm.ac.uk

**Figure:**
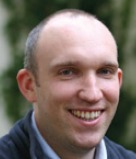
Robert Lindfield

VISION 2020 states that everyone has the right to sight. This means that, regardless of status (wealth, education, gender, impairment or other factors), everyone has the right to maximise their visual potential.

Evidence suggests, however, that many groups in society (for example women, those who are poor, or those who are disabled) are frequently unable to access eye care services. When they do, these disadvantaged groups experience poorer care despite their greater need. Providing services that are equitable – that are available and affordable to all – has been a priority for VISION 2020, and those organisations that support the initiative, since 1999.

There is limited evidence, however, that cataract surgery is reaching these groups. A recent study conducted by the London School of Hygiene and Tropical Medicine^1^ asked eye hospitals throughout the world to report the preoperative visual acuity of the next 100 cataract operations they were going to perform. Even in the hospitals in the poorest countries, where the prevalence of cataract blindness (and hence the need for surgery) was high, only 40% of operations were on people who were blind from cataract. Instead, the hospitals were offering surgery to people who were not yet blind, which is hard to justify considering that there were so many people who were blind and who needed an operation more urgently.

**Figure:**
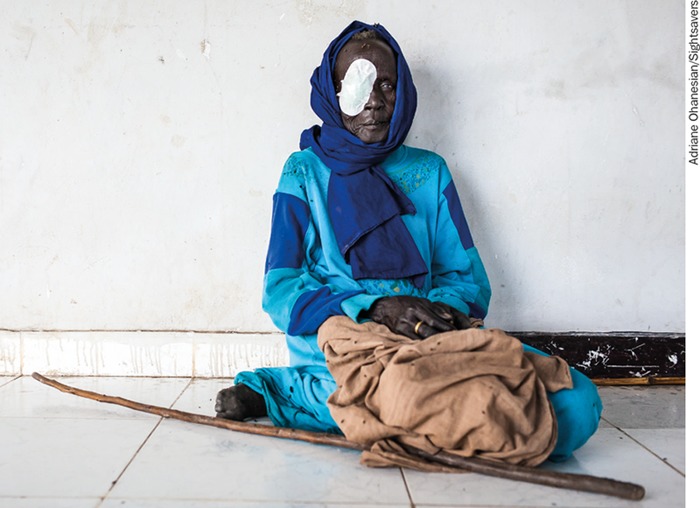
A woman rests in the hallway after her cataract operation, conducted at a clinic in South Sudan as part of an outreach programme.

Tackling unequal access to cataract surgery for women has been a priority for VISION 2020 since its inception. Unpublished data from three ophthalmology centres in Uganda revealed that, of the 2,800 cataract operations performed in 2013 for which information on gender were available, 50.2% were on women. The Uganda Bureau of Statistics estimates that 56% of Ugandans aged over 50 are women. This suggests that women are not accessing cataract surgery to the same degree as men in this setting, and is a finding that is repeated elsewhere.

**ABOUT THIS ISSUE**

**Figure:**
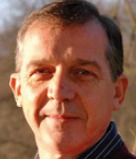
Allen Foster

Co-director: International Centre for Eye Health, London, UK.

Cataract remains the number one cause of bilateral blindness in the world. This is despite improvements in surgical technique resulting in better visual outcome and – using a variety of cost containment and income generation activities – attempts to lower the cost of surgery.

There are many good examples of the delivery of high volume, good quality and low cost cataract surgical services throughout the world. Unfortunately, however, there are also many places that have low volume, expensive cataract services, with less than optimal outcomes for patients.

A critical question, then, is how to transform a system with ineffective and inefficient delivery of cataract services into one with effective (good results) and efficient (good use of resources) delivery? This requires providers to ensure that they are delivering efficient eye care services with high quality surgery at a reasonable cost, together with activities in the community to create demand and overcome barriers to access.

This issue of the *Journal* includes case studies from Asia and Africa, together with articles on best practice, to try and assist readers to improve the quantity and quality of existing cataract services, while realising that each situation is different and has its own challenges, but also its own opportunities for good and innovative solutions.

The results from the most recent Rapid Assessment of Avoidable Blindness (RAAB) surveys suggest a similar finding. Almost uniformly, these surveys reveal that the number of men who have had cataract surgery is higher than the number of women, despite the fact that there are more women in the older age groups. This suggests that men find it easier to use (and pay for) the services that are provided.

There is very little information about poverty and access to cataract surgery. We know that cataract surgery contributes to a reduction in poverty, but it is also thought that people who live in poverty are less likely to access services. Unfortunately, we do not collect much information on these people, and few studies have focused on the best ways to reduce their barriers to seeking care.

So why does it appear that we are failing to address inequity in cataract surgery services?

It is recognised that there is a drive towards financial sustainability within hospitals. This is often achieved by asking wealthy patients to supplement the cost of operations for those who are too poor to pay. For this to work, wealthy patients must be attracted to local hospitals and there are a variety of successful tactics to achieve this. At the same time, anecdotal evidence suggests that hospitals are also cutting costs by closing down outreach programmes that target hard-to-reach groups. This implies that many hospitals are investing in attracting wealthy people at the expense of treating those in greatest need. This is borne out by the LSHTM study.^1^

Creating financial sustainability is good, as is cross-subsidisation for cataract operations for people who are unable to afford them. However, unless hospitals make a conscious effort to target hard-to-reach groups, inequity will widen.

The World Health Organization (WHO) has recognised that people from deprived groups find it difficult to make use of services, predominantly because people are usually expected to pay something towards the cost of their own treatment. It is thought that this ‘out-of-pocket spending’ on health by people in low-income countries leads 100 million people into extreme poverty every year.

Cost also prevents people who are already in poverty from accessing services.^2^ A recent study assessing the impact of cost on the uptake of cataract services in Nigeria^3^ indicated that the indirect costs of coming for cataract surgery (including transport, food and the cost of bringing an accompanying person) is nearly double its direct cost.

To address these issues, the WHO has introduced the concept of ‘universal health coverage’, whereby health systems support equitable access by making services affordable whilst ensuring that they are of high quality. The main focus of universal health coverage is to ensure that out-of-pocket spending is kept as low as possible and that no one enters poverty as a result of health costs, or is excluded from health care because of costs.

The United Nations has adopted a resolution on universal health coverage that urges governments to move towards providing all people with access to affordable, good quality health care services.

As members of the eye health community, it is our responsibility to support this resolution and to promote action towards universal access to eye health. This means advocating with colleagues in other health sectors and with governments, non-governmental organisations and corporations to make sure that no one is excluded from essential eye care because of their age, gender, ability or socio-economic status.

This is the only way to ensure that people with the most need, including those from marginalised groups, have access to affordable, high quality cataract surgery, allowing them to maximise their visual potential and achieve the goal of VISION 2020.
